# Ontario Veterinary College

**Published:** 1895-09

**Authors:** 


### ONTARIO VETERINARY COLLEGE.

No changes will be made in the faculty the coming year. The session commences October 16, 1895, and ends the latter part of March, 1896, a period of five and a half months, during which time the various examinations are conducted. We are sorry to note the continuation of the feature that those who fail to pass the matriculation examination will be allowed to pursue their studies, specially so when the extent of the examination is covered by reading, writing, and spelling. The publishing of the list of examination questions is a good feature, and imparts a general idea of the scope of instruction. The insertion of promiscuous advertisements in the college catalogue rather detracts from its scientific aspect.

